# Banded Roux-en-Y gastric bypass in patients with super morbid obesity (BRandY-study): protocol of a cohort study with 10 year follow-up

**DOI:** 10.1186/s12893-020-00784-x

**Published:** 2020-06-05

**Authors:** M. M. Romeijn, W. K. G. Leclercq, A. A. P. M. Luijten, L. Janssen, F. M. H. van Dielen

**Affiliations:** Obesity Center Máxima, Máxima Medical Center, Department of Surgery, Eindhoven/Veldhoven, the Netherlands

**Keywords:** Super morbid obesity, Bariatric surgery, Roux-en-Y gastric bypass, Non-adjustable gastric ring, Insufficient weight loss, Weight regain

## Abstract

**Background:**

Weight loss outcomes after bariatric surgery are less favorable in super morbidly obese patients (BMI ≥50 kg/m^2^). Non-response, either defined as insufficient weight loss or weight regain after initial successful weight loss, is a matter of serious concern in these patients. The primary banded Roux-en-Y gastric bypass has shown promising results regarding weight loss in the bariatric population. However, up to now, long-term comparative data about the banded and non-banded bypass in superobese patients is lacking. The aim of this study is to assess the added value of the banded Roux-en-Y gastric bypass in superobese patients on long-term weight loss outcomes.

**Methods:**

This single center study will evaluate superobese patients who receive a non-banded Roux-en-Y gastric bypass (NB-RYGB) and a banded Roux-en-Y gastric bypass (B-RYGB). Data from the NB-RYGB group will be collected in retrospect, while data from the B-RYGB group will be collected prospectively. When performing a B-RYGB, a 7.0–8.0 cm silastic ring (MiniMizer®) will be placed proximal to the gastrojejunostomy. The main outcomes of this study are weight loss and non-response during a 10 year follow-up period. Secondary outcomes are reduction of obesity related comorbidities and medication, (ring-related) morbidity and mortality, complications, re-operations, patient satisfaction and health-related quality of life. A total of 142 patients will be included in this study.

**Discussion:**

This study will help establish the clinical utility of the B-RYGB in superobese patients.

**Trial register:**

NL8093. Registered 15 October 2019 - Retrospectively registered on the Dutch Registry of Clinical trials, www.trialregister.nl

## Background

The last three decades have shown that the prevalence of patients with super morbid obesity, classified as Body Mass Index (BMI) ≥50 kg/m^2^, increases faster than patients with morbid obesity [1]. Although it is well established that bariatric surgery is an effective treatment for these patients, weight loss outcomes are less beneficial in patients with super morbid obesity. Where morbidly obese patients lose an average of 67% excess weight loss (EWL) in the first 2 years after Roux-en-Y gastric bypass (RYGB) [2], super obese patients lose between 48 and 59% [3, 4]. Furthermore, morbidly obese patients reach their maximal weight loss 1.9 years after surgery, while super obese patients reach this point after 2.2 years [5, 6].

Another concern is that the rate of non-response after bariatric surgery seems to be more pronounced in superobese patients [7]. Non-response can be defined as either insufficient weight loss (primary non-response), or regain of an excessive amount of weight after initial successful weight loss (secondary non-response) [9]. Based on recent literature, 12–16% of superobese patients do not achieve successful weight loss 2 years after RYGB, classified as total body weight loss (TBWL) < 20% and EWL < 50%, respectively [8]. Long term studies have also shown that superobese patients gain 5.2 BMI points 5 years after RYGB [7]. Ten years after RYGB 58% of superobese patients have strikingly returned to a BMI above 35 kg/m^2^ [5] not to mention the comorbidities that may have reoccurred.

Due to these high rates of non- response, variations to the gastric bypass have gained interest. This includes band placement, lengthening of the biliopancreatic limb and revision of the gastric pouch and/or stoma [5, 10, 11]. The gastric band controls pouch and stoma size and is suggested to prevent further dilatation thereby inducing weight loss and preventing non-response [12, 13]. Another advantage is that it possibly minimizes dumping syndrome since it prevents rapid emptying of the pouch to the jejunum. Potential disadvantages are food intolerance and complications like band erosion, slippage and small bowel obstruction which may result in additional surgery [6].

As a primary operation, the banded bypass has shown to be reasonably efficacious in (super) morbidly obese patients. Based on two large systematic reviews, the banded bypass improved EWL with 15–20% 3–5 years after surgery [2, 6]. It also resulted in 11.2% less non-response, expressed as a regain of more than 5 BMI points 5 years after surgery [14]. Unfortunately, less extensive studies have been performed solitary in the group of superobese patients. So far, these studies found 10% more EWL 2 years after banded bypass [3] and no differences in non-response after 5 years [15]. These studies are limited by the low number of included patients, lost to follow-up and short follow-up period.

Since non-response in superobese patients is a serious concern, there is increasing need for clinical guidance. Studies about the banded bypass in these patients have shown promising results but lack long-term comparative data. This study was set out to assess the additive value of a banded RYGB, by using the MiniMizer®, on non-response in patients with super morbid obesity. It is hypothesized that the MiniMizer® hampers a large food bolus to enlarge the pouch and stoma in time, thereby preventing secondary non-response. Furthermore, it may delay food passage through the pouch resulting in decreased food intake, thereby preventing primary and secondary non-response.

## Methods/Design

### Study cohort

This single center cohort study will be performed at the Máxima Medical Center, a European bariatric center of excellence. In order to compare outcomes of patients that received a banded Roux-en-Y gastric bypass (B-RYGB) with the non-banded Roux-en-Y gastric bypass (NB-RYGB), both retrospective and prospective collected data will be analyzed. The retrospective cohort consists of patients that were operated between January 1st 2017 and November 30st 2019. At December 2019, the B-RYGB was introduced in our centre as standard of care. From that point on, data is collected prospectively.

### Patient selection

Patients will be included if a NB-RYGB or B-RYGB was performed because of a preoperative BMI of 50 kg/m^2^ or greater (1) under the conditions that there were no preoperative signs of severe psychiatric and psychological disorders assessed by a medical psychologist (2) and that they were committed to attend the follow-up appointments in our center (3). Patients will be excluded if they already underwent a bariatric procedure such as gastric banding.

### Study outcomes

The main outcomes of this study are weight loss and non-response within 10 years after surgery. Non-response is defined as either primary (insufficient weight loss) or secondary (significant weight regain), as described below. Secondary outcomes are reduction of obesity related comorbidities and medication, (ring-related) morbidity and mortality, complications, re-operations, patient satisfaction and health-related quality of life during a 10 year follow-up period (Table 1).

**Table 1:** Overview of data collection

	Baseline	30 days	1 year	1.5 year	2 years	3 years	4 years	5 years	6 years	7 years	8 years	9 years	10 years
Demographics	**x**												
Co-morbidities & medication	**x**		**x**	**x**	**x**	**x**	**x**	**x**	**x**	**x**	**x**	**x**	**x**
Morbidity and mortality					**x**			**x**					**x**
Complications and re-operations		**x**			**x**			**x**					**x**
Weight loss			**x**	**x**	**x**	**x**	**x**	**x**	**x**	**x**	**x**	**x**	**x**
Primary non-response				**x**									
Secondary non-response					**x**	**x**	**x**	**x**	**x**	**x**	**x**	**x**	**x**
Health related quality of life	**x**		**x**			**x**		**x**					**x**
Patient satisfaction						**x**		**x**					**x**

### Standard pre- and postoperative care

All patients are screened for surgery by a multidisciplinary team. To qualify for bariatric surgery IFSO-criteria are used [16]. Two weeks prior to surgery patients are restricted on a very low-calorie diet (Modifast) containing 450–500 kcal/day in order to reduce weight and liver volume. The presence of Helicobacter Pylori is routinely assessed by serological tests and, if present, patients are treated with standard triple therapy. To rule out a hiatal hernia, barium swallow study and/or upper endoscopy are performed when a patient reports complaints like gastro-esophageal reflux. All patients are regularly monitored in the outpatient clinic by members of the multidisciplinary team for a period of 5 years. Regular blood testing is performed including hemoglobin, iron, ferritin, transferrin, albumin, calcium, folic acid, vitamin D, vitamin B (B1, B6, B12) and zinc. Furthermore, HbA1c is assessed in patients with diabetes mellitus.

### Operative technique

Bariatric surgery involves laparoscopic RYGB with the use of a modified circular stapling technique described by Dillemans [17]. Surgery is performed by three bariatric surgeons with over 10 years of experience in primary and revisional bariatric surgery. Roux-en-Y is constructed with a pouch volume of 15-30 cc, an estimated biliopancreatic limb of 70-100 cm and an estimated alimentary limb of 130-150 cm in all procedures. The integrity of the anastomosis is tested with methylene blue and air leakage. Laparoscopic placement of the gastric ring (MiniMizer® Gastric Ring, Bariatric Solutions) is performed according to the manufacturer’s instructions [18]. A small perigastric tunnel is made in which the silastic ring is entered. The ring is placed around the pouch, 4–6 cm from the gastroesophageal junction or 2 cm proximally to the gastrojejunostomy. The ring is closed at either 7.0, 7.5 or 8.0 cm depending on the size of the gastric pouch. By inserting a gastric tube of 34-French before locking the ring, it is tested whether the ring is not to thigh. After locking, the tip of the ring is cut and removed. The ring is fixed by 2 non-absorbable stitches placed at the gastric wall. Petersen space is routinely closed, while the mesodefect of the enteroenterostomy is only closed on indication. For closure of defects, a non-absorbable suture is placed in a running fashion. In case of a hiatal hernia, a laparoscopic crural repair is performed with sutures and/or mesh placement. This repair is performed during the same procedure as the gastric bypass. If the hernia is too large and therefore inoperable, the patient does not receive a MiniMizer®.

### Data collection

Data will be collected by the coordinating investigator at multiple times: baseline (pre-operatively), at 30 days, 1, 1.5, 2, 3, 4, 5, 6, 7, 8, 9 and 10 years after surgery. Data will be recorded in an online case record form only accessible by logging in to a website (https://myresearchmanager.com). Data includes patient demographics, comorbidities and medication use, operating time, length of hospital stay, (ring-related) morbidity and mortality, complication and reoperations. Comorbidities that are taken into account are hypertension, dyslipidemia, type 2 diabetes, sleep apnoea syndrome, osteoarthritis and gastroesophageal reflux.

### Assessment of non-response and weight loss

Weight is noted at baseline and at each follow-up visit. Weight loss is described as the percentage of total body weight loss (%TBWL) and excess weight loss (%EWL). %TBWL is calculated as follows: ((preoperative weight – lowest weight) / preoperative weight)) × 100. %EWL is calculated as follows: ((preoperative weight − lowest weight) / (preoperative weight − ideal body weight based on an BMI of 25 kg/m^2^)) × 100. Patients are categorized as primary non-response (1NR) if the patients’ %TBWL is less than 15% within 18 months after surgery. Patients are categorized as secondary non-response (2NR) if they gain more than 20% of lost weight after an initial TBWL of 15% 24 months after surgery and beyond [19].

### Assessment of quality of life

Health related quality of life (HRQOL) is measured using the RAND-36 questionnaire. This questionnaire contains 36 questions and 9 scales: emotional role functioning, physical role functioning, social functioning, physical functioning, vitality, mental health, bodily pain, general health perceptions and health change. Two subtotal scores can be calculated: physical health summary and mental health summary. The total score ranges from 0 to 100 where a higher score represents a higher quality of life. The questionnaire is validated in the obese population (test-rest reliability *r* = 0.94; internal consistency, Cronbach’s alpha 0.96) [20].

### Assessment of patient satisfaction

Patients’ satisfaction is measured using the Net Promotor Score (NPS) [21] and is based on the question: “How likely is it that you would recommend our hospital to a friend or colleague?” Patients can give an answer ranging from 0 (‘not at all likely’) to 10 (‘extremely likely’). The assumption is that patients scoring a 9 or a 10 will give positive word-of-mouth advertising; they are called ‘promoters’. Patients answering 7 or 8 are considered indifferent (‘passives’). Finally, patients answering 0–6 are likely to be dissatisfied and are labelled as ‘detractors’. The NPS is then calculated using the formula: % of Promoters - % of Detractors. The resulting score can range from − 100 to + 100. Based on global NPS standards, any score above 0 would be considered “good.”

### Sample size

The sample size calculation is based on a study that showed a difference of 8.8% in EWL between the group of NB-RYGB and B-RYGB 5 years after surgery [14]. Since this data is based on a population of morbidly obese patients, we aim to detect a difference of ≥10% EWL in the population of superobese patients. An alpha of 0.05 is used with a power of 0.80 (two-sided test). In total 10% dropouts will be expected based on no-show. A sample set of 142 patients, 71 per arm is needed to achieve statistical relevance and prevent ambiguity.

### Statistical analysis

Differences in weight loss, non-response, comorbidities, morbidity, mortality and complications between the two groups are analyzed using an independent samples t-test (or Mann-Whitney U in case of non-normal distribution) and chi-square test (or Fisher exact in case of small numbers). The association between non-response rates (outcome) and surgical technique (exposure) is further analyzed using a multivariate logistic regression. First, known risk factors of non-response (e.g. baseline body mass index, age at surgery, gender [22]) and also other study variables (demographics, comorbidities, complications) are tested as possible confounders in univariate regression analyses (with outcome non-response). Variables that are associated with the outcome (*p* < 0.1) are entered as covariates in the multivariate regression analysis. A *p*-value < 0.05 is considered statistically significant. SPSS will be used for statistical analysis (IBM Corp, Released 2013, IBM SPSS Statistics for Macintosh, recent version).

## Discussion

This study will assess if the banded RYGB, which is designed for controlling pouch and stoma size, can improve weight loss and prevent long-term non-response in the superobese patient. This study seeks evidence for the establishment of the clinical utility of the banded RYGB in superobese patients.

## Data Availability

The datasets used and/or analysed during the current study are available from the corresponding author on reasonable request.

## Abbreviations

BMI: Body Mass Index
B-RYGB: Banded Roux-en-Y gastric bypass
EWL: Excess Weight Loss
HRQOL: Health related quality of life
NPS: Net Promotor Score
1NR: Primary non-response
RYGB: Roux-en-Y gastric bypass
2NR: Secondary non-response
TBWL: Total body weight loss
